# Latent Membrane Protein 1 Is Dispensable for Epstein-Barr Virus Replication in Human Embryonic Kidney 293 cells

**DOI:** 10.1371/journal.pone.0022929

**Published:** 2011-08-11

**Authors:** Vicki Geiser, Ellen Cahir-McFarland, Elliott Kieff

**Affiliations:** The Department of Medicine, Division of Infectious Disease, Brigham and Women's Hospital and the Department of Microbiology and Molecular Genetics, Harvard Medical School, Boston, Massachusetts, United States of America; University of Minnesota, United States of America

## Abstract

Epstein Barr Virus (EBV) replicates in oral epithelial cells and gains entry to B-lymphocytes. In B-lymphocytes, EBV expresses a restricted subset of genes, the Latency III program, which converts B-lymphocytes to proliferating lymphoblasts. Latent Membrane Protein 1 (LMP1) and the other Latency III associated proteins are also expressed during virus replication. LMP1 is essential for virus replication and egress from Akata Burkitt Lymphoma cells, but a role in epithelial cell replication has not been established. Therefore, we have investigated whether LMP1 enhances EBV replication and egress from HEK293 cells, a model epithelial cell line used for EBV recombinant molecular genetics. We compared wild type (wt) and LMP1-deleted (LMP1Δ) EBV bacterial artificial chromosome (BAC) based virus replication and egress from HEK293. Following EBV immediate early Zta protein induction of EBV replication in HEK293 cells, similar levels of EBV proteins were expressed in wt- and LMP1Δ-infected HEK293 cells. LMP1 deletion did not impair EBV replication associated DNA replication, DNA encapsidation, or mature virus release. Indeed, virus from LMP1Δ-infected HEK293 cells was as infectious as EBV from wt EBV infected HEK cells. Trans-complementation with LMP1 reduced Rta expression and subsequent virus production. These data indicate that LMP1 is not required for EBV replication and egress from HEK293 cells.

## Introduction

Epstein-Barr Virus (EBV), like most human herpes viruses, spreads through saliva and is believed to initally replicate in the oropharyngeal epithelium (for review see [1]). Circulating naïve B cells are infected early in primary human infection. In these cells, a latency III (LTIII) program of virus gene expression promotes infected B cell replication, and differentiation into memory B cells, which are the reservoir for reactivated EBV replication and infection of neighboring epithielial cells [2], [3] [4], [5].

Because EBV LTIII infection transforms B lymphocytes into immortal lymphoblast cell lines (LCLs), in vitro, the genetics and biochemistry of LTIII infection have been extensively studied. In particular, Latent Membrane Protein 1 (LMP1) induces NFκB, has Ras-like transforming effects on cells, and is expressed in most EBV-related malignancies including Post-transplant lymphoproliferative disorders, Hodgkin's Disease, and Nasopharyngeal Carcinoma (for review see [1]). LMP1-mediated NFκB activation is required for B cell growth and survival [6], [7], [8], [9], [10].

LTIII proteins are also expressed during replication in both B and epithelial cells. In Akata Burkitt tumor cells, LMP1 expression is detected 8 hours after IgG stimulation, prior to EBNA2 expression, consistent with Rta stimulation of the LMP1 promoter [11], [12]. Within 48 hours of IgG crosslinking, LMP1 and other LTIII proteins increase substantially [12], [13]. EBNA2 and LMP1 are also expressed with EBV replication in differentiating epithelial, as is most evident in foci of EBV replication in the oropharyngeal or lingual epithelia of immune compromised people [14], [15].

Although expressed, relatively little is known about the requirement for LTIII EBV gene expression for replication in epithelial cells [12], [13], [14], [16]. LMP1 has been shown to have an effect on replication in B lymphoblasts, but an effect in epithelial cells has not been established. In the Akata Burkitt lymphoma, LMP1 is important for EBV maturation and egress [17]. An Akata virus mutant, deleted for LMP1 accumulates empty and filled capsids in the nuclei, with little evidence for enveloped virus maturation and release [17].

LMP1 activation of NFκB or of MAP kinases may be important to support virus replication in epithelial cells. LMP1 activates NFκB and TRAF1 expression in Oral Hairy Leukoplakia lesions [14] and induces cell gene expression late in virus replication [12]. However, LMP1 mediated NFκB or MAP kinase activation requires LMP1 transmembrane domains 1–6 [18], [19], whereas expression of LMP1 deleted for transmembrane domains 1–4 enabled EBV egress in LMP1 deficient Akata cells [17], consistent with an different role for LMP1 in virus maturation.

We have therefore compared wt and LMP1-deleted virus for replication in HEK293 cells, an Adenovirus E1A and E1B transformed human embryonic kidney cell line that supports EBV replication and has been extensively used for recombinant EBV molecular genetics [20]. HEK293 are similar to primary epithelial cells, in keratin and kinase expression [21], [22].

## Results

### Construction of an LMP1-deleted EBV BAC, LMP1Δ BAC

**Figure 1:**
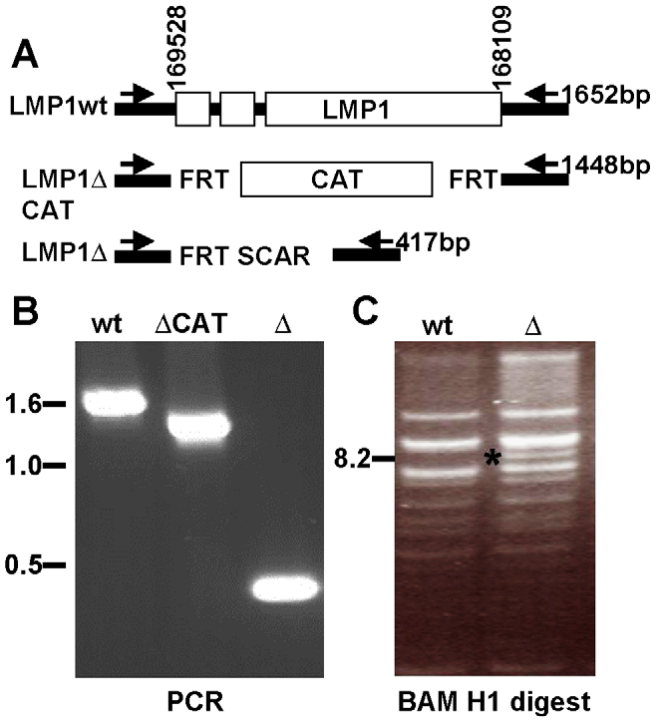
Construction of the LMP1Δ Bacterial artificial chromosome. (A) Schematic of wildtype and resulting LMP1Δ viruses. EBV coordinates according to B958 numbering. Primers for PCR screening indicated. (B) PCR across the LMP1 loci of the indicated BAC. (C) BAMH1 digest indicates the wt and LMP1Δ BAC genomes have no gross deletion. A unique 8.2 kB fragment (*) corresponds to the expected size of the BAM N fragment harboring the LMP1 deletion.

The LMP1 ORF was replaced with the chloramphenicol acetyl transferase (CAT) gene flanked by FLP recombinase target (FRT) sites, using lambda red mediated homologous recombination and wt EBV-BAC (MD1 [23]) (LMP1ΔCAT, Fig. 1A). CAT was subsequently removed by FLP recombinase, leaving a single 84-bp FRT scar sequence in place of the LMP1 ORF (LMP1Δ). Successful recombinants were identified by PCR, using primers that flank the LMP1 ORF. PCR amplification of wt EBV-BAC, LMP1ΔCAT, and LMP1ΔBAC DNA yielded specific products at 1652 bp, 1448 bp and 417 bp, respectively (Fig. 1B). BamH1 digestion of the LMP1Δ DNA revealed the expected novel 8.2 kB LMP1 deleted Bam N fragment, which is 1.4 kb smaller than the wt 9.6 kb Bam N fragment (Fig. 1C).

Wt and LMP1Δ BACs were introduced into HEK293 cells by transfection. Wt and LMP1Δ BAC HEK293 cell clones were isolated by selection for puromycin resistance, which is expressed from the BAC F plasmid. PCR of 5 widely separated EBV genome sites in BamU, EBNA3C, BcLF1, BALF5, BNFL2b confirmed these sites in the infected cell clones. HEK293 cell clones without spontaneous EBV replication, which could be induced to replicate by Zta expression, and were identifiable by Zta-induced surface gp350 expression, were used for further studies. A wt and an LMP1Δ HEK293 EBV clone were extensively characterized.

### LMP1 is not required for immediate early or early protein expression in HEK293 cells

**Figure 2:**
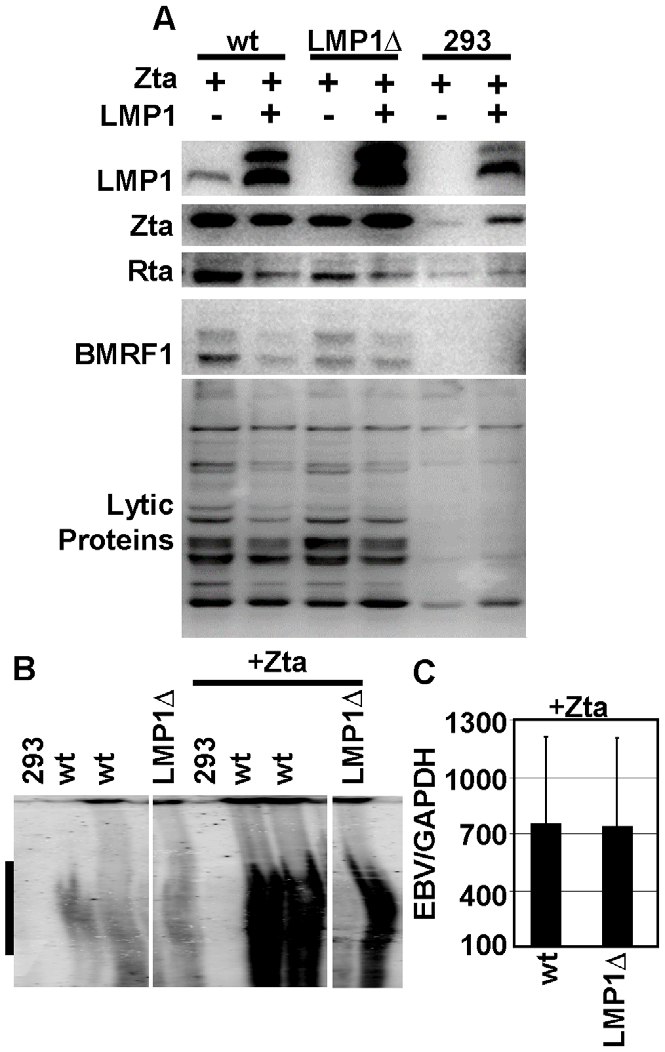
LMP1Δ BAC replicates to wildtype levels in HEK293 cells. (A) Western blot analysis of 293 cells with the indicated BAC 4 days after the virus was induced to replicate with Zta or Zta and LMP1 expression. Rta, BMRF1 and lytic protein expression was induced to the same extent in wt and LMP1Δ cells. LMP1 transcomplementation inhibited Rta expression. (B) Zta induces virus DNA replication in wt and LMP1Δ HEK293 cells, Gardella gel analysis, linear genomes marked by black bar. (C) Zta-induced replication is unaffected by LMP1 deletion; .EBV (BALF5) per GAPDH copies of intracellular virus was normalized to the percentage of gp350+ cells.

Following transfection of HEK293-EBV clones with Zta, or Zta and LMP1, cells were cultured for 4 days and examined for LMP1 and Zta expression. LMP1 expression was detected in cells harboring the wt EBVBAC but not in cells harboring LMP1Δ BAC or control HEK293 cells, verifying the knockout phenotype (Fig. 2A). As expected, LMP1 levels were higher in the LMP1 transcomplemented cells. LMP1 expression induced higher level Zta expression in control 293 cells by activating NFκB, which increases SV40 promoted Zta expression (Figure 2A) [24], [25]. This effect was masked by Zta expression from the replicating EBV genomes in wt and LMP1Δ BAC infected cells (Figure 2A). Notably, Zta levels were similar in wt and LMP1Δ BAC cells under all conditions. To determine if LMP1 affects replication associated protein expression in 293 cells, we compared immediate-early Rta, early BMRF1, and bulk human antibody detected replication protein expression in wt and LMP1Δ BAC infected cells. Rta and BMRF1 were similarly induced by Zta in both wt and LMP1Δ cells (Figure 2A). Over-expression of LMP1 repressed Rta consistent with earlier findings and, as a consequence, slightly decreased BMRF1 levels (Figure 2A). Overall EBV replication associated protein expression as detected by human serum blots paralleled Rta and BMRF1 levels and was similar in wt and LMP1Δ cells (Fig. 2A, bottom panel). These results indicate that Zta induced LMP1Δ BAC HEK293 cells expressed EBV replication associated proteins at levels comparable to Zta induced wt EBVBAC.

### LMP1 is not required for EBV DNA replication in HEK293 cells

EBV DNA accumulation in Zta-induced LMP1ΔBAC versus wt EBVBAC infected cells was assessed using Gardella gels to separate latent infection associated episomes from Zta induced linear (replicated) EBV DNA [26]. Wt EBV BAC and LMP1Δ BAC infected HEK293 cells had similar low levels of linear EBV DNA (Figure 2B). Further, Zta induced similar high levels of linear EBV genomes in both wt and LMP1Δ BAC infected HEK293 cells (Fig. 2B). Thus, DNA replication was not substantially affected by the LMP1 deletion. EBV genome copy number in Zta-induced wt BAC versus LMP1Δ BAC infected HEK293 cells was evaluated by qPCR for EBV BALF5 DNA versus cell GAPDH, after correction for the percentage of cells that expressed surface gp350. Wt EBV BAC and LMP1Δ BAC infected HEK293 cells had 7–10 EBV genomes per GAPDH DNA. Zta transfection increased EBV/GAPDH ratio to 752 for wt and 737 for LMP1Δ BAC infected HEK293 cells indicating that wt BAC and LMP1Δ BAC infected HEK293 cells are similarly responsive to Zta-induced DNA replication (Figure 2C). LMP1 transcomplementation along with Zta expression had similar negative effects on wt BAC and LMP1Δ BAC infected HEK293 gp350+ cell fraction and EBV DNA copy number.

### LMP1 is not required for EBV DNA encapsidation, egress, and infectivity

**Figure 3:**
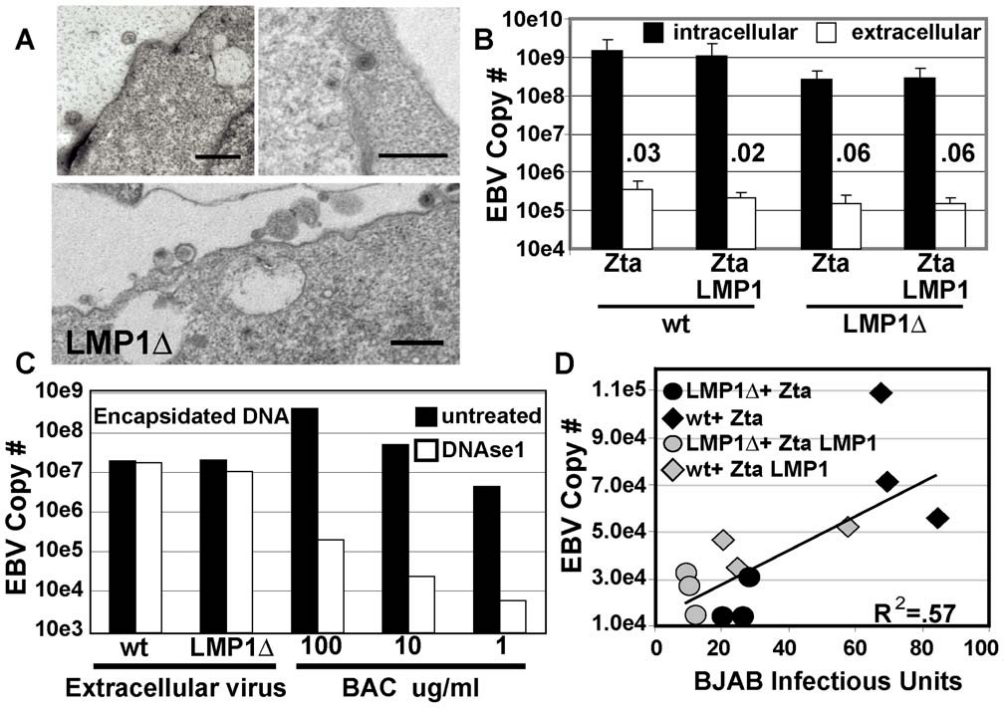
LMP1Δ virus shows no defect in egress, encapsidation or infectivity. (A) 3 representative Transmission Electron Micrographs of HEK293 cells producing LMP1Δ virus, black bars = 500 nm. (B) LMP1Δ virus shows no defect in egress from HEK293 cells. Quantization of intracellular and extracellular EBV DNA by qPCR for BALF5 (not normalized to gp350). Ratio between the two is presented (n>5, standard deviation is shown). (C) Extracellular LMP1Δ virus is encapsidated normally. Extracellular virions were digested with or without DNAse 1 and quantitated by BALF5 qPCR. BAC DNA produced in E. Coli served as a DNAse sensitive control (n = 2). (D) LMP1Δ virus is equally infectious as wt virus. BJAB was infected and colony formation after puromycin selection was determined at 4–6weeks post infection. BJAB infectious units are plotted as a function of EBV BALF5 copy number in the supernatants (n = 3). The relationship between EBV copy number and BJAB infectious units is linear, R^2^ = .57. Open symbols, LMP1 was transfected with Zta, closed symbols, Zta alone.

Since LMP1 deleted Akata EBV infected Akata cells were deficient in post nuclear virus morphogenesis and mature virus release [17], transmission electron micrographs (TEM) of Zta-induced LMP1Δ BAC infected HEK293 cells were compared to Zta induced wt BAC infected HEK293 cells. No differences were observed. In both instances, infected cell nuclei had scattered unfilled capsids and most capsids near the nuclear membrane were filled. Enveloped virus was found between the inner and outer nuclear membrane and in cytoplasmic vesicles. Capsids in various stages of cytoplasmic and plasma membrane envelopment, and mature enveloped extracellular virus were readily evident (Figure 3A). Thus, Zta-induced wt EBV BAC and LMP1Δ BAC infected cells were similar in normal capsid and enveloped virus morphogenesis and extracellular virus appearance.

The efficiencies of EBV egress from Zta-induced LMP1Δ BAC and wt BAC infected HEK293 cells were quantified by assessing cell associated and supernatant EBV DNA, using qPCR for EBV BALF5 DNA. Zta-induced LMP1Δ BAC and wt BAC infected HEK293 cell EBV genome copy number varied with Zta-induced gp350 expression levels (Figure 3B). As expected, intracellular EBV DNA levels were nearly 4 logs higher than extracellular enveloped virus associated DNA levels. Most importantly, Zta-induced LMP1Δ BAC and wt BAC infected HEK293 cell intracellular or extracellular EBV genome copy numbers were to within 5 fold of each other and there was less than 2-fold difference in the amount of extracellular DNA. Further, LMP1 expression in trans had little or no effect on intra- or extra- cellular EBV DNA levels (Figure 3B). Moreover, the ratio of extracellular to intracellular DNA copies was .03% and .06% for wt and LMP1Δ genomes. In sum, the efficiency of egress was not LMP1 dependent (Figure 3B).

To evaluate whether EBV genomes detected in culture supernatant are encapsidated or enveloped, DNAse1 resistance was assessed. Most extracellular DNA was DNAse resistant, whereas control BAC DNA was highly DNAse sensitive. Overall, 70% of wt and 55% of LMP1Δ extracellular virus genomes were DNAse 1 resistant (Figure 3C). Thus, LMP1Δ BAC was not deficient in EBV egress and LMP1 trans-complementation did not increase extracellular virus DNA.

Since wt and LMP1Δ BAC were similar in replication and release of extracellular virus, we proceeded to evaluate whether the virions were equally infectious. Because LMP1Δ virus does not transform peripheral blood B cells ([7] and data not shown), we compared wt and LMP1Δ EBV in infection of BJAB, an EBV-negative Burkitt lymphoma cell line. Efficiency of EBV infection was determined by measuring EBV DNA copy number in the supernatants used to infect BJAB cells and by the conversion of BJAB cells to puromycin resistance. BJAB infection was proportional to DNA copy number for both wt and LMP1Δ viruses (Fig. 3D, R^2^ = .57). LMP1 expression in trans did not increase the infectivity of either wt or LMP1Δ EBV (filled vs. open symbols, Figure 3D). Thus, LMP1Δ virus produced in HEK293 cells was not deficient in replication, egress, or infectivity.

## Discussion

In this study, we have found LMP1 deleted EBV to not differ from wt EBV in HEK293 cell replication, replication associated protein expression, DNA replication, encapsidation, egress, or infectivity. Since a role for LMP1 in EBV egress from infected Akata Burkitt tumor cells was previously reported [17], we carefully evaluated if LMP1Δ virus produced in HEK293 was impaired at this step. We observed virions in the cytoplasm of LMP1Δ cells. Likewise, the rate of egress and presence of infectious virions was not affected by LMP1 deletion. Therefore, the effect of LMP1 deletion on virus replication differs if the virus is generated from Akata or HEK293 cells.

The requirement for LMP1 in replication and release from Akata cells may reflect a difference between B-cells and HEK293 cells. In the Akata study, a truncated LMP1 that does not activate NFκB partially complemented egress [17]. D1 LMP1 can interact with IRF7 [27] and IRF7 may be important for EBV replication in B-cells. Alternatively an LMP1 effect in replication may not be required in 293 cells, which express Adenovirus E1A and E1B. LMP1 and E1A and E1B may overlap in effects on cell growth or DNA damage response pathways that may accompany virus replication in a B-Lymphoma cell background [28]. Alternately, the difference between the effect of LMP1 deletion may be due to the virus genomes used. The Akata genome is wild-type, whereas our study is based on a B958 BAC which is deleted for LF1, 2, and LF3 [29]. We cannot exclude the possibly that LMP1 may be essential for modifying an LF1, 2, or 3 function or may be cell type specific in affecting egress.

Although we have found no requirement for LMP1 expression in HEK293 cells, LMP1 may have important effects in differentiating or differentiated epithelial cells. Normal epithelium undergoes a tightly regulated differentiation pathway. Basal cell attached to a basement membrane may confer stem cell characteristics which precede differentiation into spinous, granular, or apical epithelial cells. Herpes, Adeno, and Human Papilloma virus DNA virus replication is coupled to epithelial cell differentiation. EBV and KSHV enter lytic replication in the suprabasal keratinocytes in organo-raft cultures or in vivo [30], [31]. EBV replication is also coupled to differentiation in Oral Hairy Leukoplakia lesions. The homologous Rhesus lymphocryptovirus DNA and Zta protein expression are found in the Granular layer whereas VCA is expressed at the surface layer of OHL lesions in macaques [32]. LMP1 interacts with TRAF1 and activates NFκB in human OHL [14]. LMP1 can also effect epithelial differentiation and promote epithelial to mesenchymal transition [33], [34]. Thus, LMP1 may have an important role in replication in normal human epithelium.

## Materials and Methods

### Cells and virus

HEK293 (ATCC CRL1573) and BJAB [35] were maintained in DMEM or RPMI 1640, respectively, supplemented with 10% Fetalplex (Gemini), Penicillin (10 U/ml) and Streptomycin (100 mg/ml).

### Construction of the LMP1Δ EBV-BAC

The EBV BAC (MD1) consists of the B958 EBV genome with the BAC sequences inserted into the one of the EBV BamHI W repeats [36]. The BAC included F plasmid sequences that support prokaryotic replication, a chloramphenicol resistance marker for prokaryotic selection, and a Cytomegalovirus promoter-driven puromycin resistance cassette for eukaryotic selection. The LMP1 ORF was deleted by homologous recombination using lambda phage Red-mediated recombination as shown in Fig. 1A. Transient expression of lambda phage Red recombinase in E. coli containing the MD1 BAC was induced from the temperature-sensitive pKD119 plasmid, in cells electroporated with PCR-amplified DNA containing 50 nucleotides of the EBV genome upstream of the LMP1 translational start site (EBV nucleotides 169528-169479, numbering from gi:94734074; strain B95-8), a FLP recombinase target (FRT) site, the chloramphenicol resistance marker, another FRT site, and 50 nucleotides downstream of the LMP1 translational stop site (EBV nucleotides 168109–168158). Homologous recombinants were screened for chloramphenicol resistance and deletion of LMP1 by PCR, restriction digestion, and Southern blot analyses. The chloramphenicol resistance marker was removed after expression of the FLP recombinase by the pCP20 plasmid, leaving a single 84-bp FRT scar sequence in place of the LMP1 ORF. The removal of LMP1 and the chloramphenicol resistance gene was confirmed by PCR amplification of EBV BAC DNA using primers that flank LMP1 (EBV nucleotides 167987–168006 and 169626–169645).

### EBV BAC+ HEK293 cells

5×10^5^ cells HEK293 cells were co-transfected with 1.2 µg of wt or LMP1Δ EBV-BAC DNA using Effectene (Qiagen). After 48 hours, puromycin was added to the medium. Colonies were subcloned by limiting dilution. Cell lines were analyzed for the presence of the EBV BAC and for gp350 surface expression following transfection with SV40-Zta expression plasmid.

### Virions and encapsidated DNA

To induce virus replication, HEK293-EBV-BAC cell lines were transfected with 0.3 ug of Zta plasmid and 0.9 ug of pcDNA3 or pcDNA3 LMP1. After 4 days, culture media was collected, clarified by centrifugation at 1000 RPM, 4°C, for 20 min., and virus was centrifuged to a pellet at 21,500 RPM, and 4°C, for 2 h. The pellet was suspended in 100 ul RSB buffer [10 mM Tris-HCl (pH 7.4), 10 mM KCl, 1.5 mM MgCl] and a portion was treated with 1 ul DNase I (105 U/ml) at 37 C for 2 h. In parallel, EBV BAC DNA isolated from bacteria was diluted in RSB to 100 ug/ml, 10 ug/ml, and 1 ug/ml. The reactions were stopped by adding 15 ul 20 mM EDTA and incubating at 65°C for 15 min. The suspension was diluted with 2 ul 1 M Tris-Cl (pH 8.0) and 183 ul water into a TE equivalent buffer (final concentrations 10 mM Tris pH 8.0, 1 mM EDTA).

### PCR and qPCR

PCR was done with DNA extracted from EBV-BAC harboring bacteria. EBV BAC+ HEK293 or BJAB cells were lysed in PCR lysis buffer [1× PCR amplification Buffer (Taq), 1% Triton X-100, and 0.1 mg/ml proteinase K] and analyzed by PCR or qPCR. PCR was done with 5 uL of 10× PCR buffer (Invitrogen), 3 ul 50 mM MgCl2, 1 ul 10 mM dNTPs, 0.2 uM each primer, and 1 U Taq polymerase for each 50 ul reaction. To detect the deletion of the LMP1 ORF, PCR was performed on extracted DNA using LF1 and LF2 primers. After a hot start for 2 min, each PCR cycle consisted of incubations at 95°C for 30 s, 58°C for 30 s and 72°C for 2 min. qPCR was performed on HEK293-EBV cell lines or on virion lysates. Cell extracts were prepared as described above. qPCR was conducted with 12.5 uL of 2× commercial SYBR Green qPCR buffer (QuantiTect SYBR Green PCR Kit, Qiagen), 0.2 uM each primer and 2 uL template for each 25 ul reaction. EBV copy per cells was determined by qPCR using BALF5f (5′-GAGCGATCTTGGCAATCTCT-3′) and BALF5r (5′-TGGTCATGGATCTGCTAAACC-3′) primers or GAPDHf (5′-ACTTCAACAGCGACACCCACTC-3′) and GAPDHr (5′-TCTCTTCCTCTTGTGCTCTTGCT-3′) primers. After a hot start for 15 min, each qPCR cycle was incubated at 95°C for 30 s, 55°C for 30 s, and 72°C for 30 s. Detection of qPCR product followed elongation (BALF5) or after incubation at 78°C for 30 s to remove non-specific products (GAPDH).

### Gardella gels

4 days post-transfection, 5×10^6^ cells were suspended in loading buffer (15% ficoll, 0.01% bromophenol blue, 1× TBE, 1 ug RNAse) and loaded into a well of a 0.75% Gardella agarose gel (upper 1 cm of gel consists of 0.8% agarose, 2% SDS and 1 mg/ml of pronase). Wells were overlayed with lysis solution containing 2% SDS and 1 mg/ml pronase and electrophoresed at 4°C for 4 h at 15 V followed by 18 h at 120 V. DNA was transferred to a nitrocellulose membrane and detected using a P^32^ labeled EBV W repeat probe.

### Protein analyses

Post-transfection whole-cell lysates were prepared by suspending cells in 250 ul of lysis buffer [8 mM Tris/HCl (pH 8.0), 40 mM NaCl, 1 mM EDTA, 2 mg/ml iodoacetamide, 0.05 mM sodium fluoride, 10 mM sodium pyrophosphate, 1 mM sodium ortho-vanadate, 5 mg/ml leupeptin, 5 mg/ml pepstatin, 5 mg/ml antipain, 10 mM PMSF] per 100 mm^2^ plate. SDS 10% (250 ul) was added, samples vortexed, and boiled for 5 min. Protein lysates were quantified using a modified Bradford assay (DC Protein Assay, Biorad). Total cell, 30 µg, was separated by 10% SDS-PAGE and transferred to Trans-Blot nitrocellulose membrane (Bio-Rad). EBV proteins were detected by western blotting with antibodies to LMP1 (S12), Zta (Argene clone AZ69), Rta (Argene clone 8C12), or BMRF1 (Millipore clone EA-D-p52/50 R3). In other blots, human sera that recognize multiple EBV proteins were used, along with horseradish peroxidase conjugated secondary antibody (Jackson Immuno Research, West Grove, PA) and chemiflourescent detection (Perkin Elmer, Waltham, MA).

### Electron microscopy

At 4 days post-transfection with SV40-Zta expression vector, 293 cells were fixed with 2.5% glutaraldehyde for 2 h and ultra-thin sections were prepared in the Harvard Medical School core facility. Sections were treated with 1% osmium tetroxide for 1 h, dehydrated in graded ethanol, and embedded in Epon 812 mixture. Sections were stained with 2% uranyl acetate and examined under a Tecnai G^2^ Spirit BioTWIN transmission electron microscope (TEM).

### Determination of BJAB infectious units

1 ml of 1.2×10^5^/ml BJAB cells were added to 1 ml of cell-free supernatant for 1 hour, incubated at 37°C for 1 h with agitation, and 24 h post-infection, EBV infected BJAB cells were selected with 0.3 mg/ml Puromycin (1000cells/well, 96 well plate). After 4–6 weeks, the number of wells with visible outgrowth was quantified.
